# Report of Health Care Provider Recommendation for COVID-19 Vaccination Among Adults, by Recipient COVID-19 Vaccination Status and Attitudes — United States, April–September 2021

**DOI:** 10.15585/mmwr.mm7050a1

**Published:** 2021-12-17

**Authors:** Kimberly H. Nguyen, David Yankey, Peng-jun Lu, Jennifer L. Kriss, Noel T. Brewer, Hilda Razzaghi, Mehreen Meghani, Brian J. Manns, James T. Lee, James A. Singleton

**Affiliations:** ^1^CDC COVID-19 Vaccine Task Force; ^2^Department of Health Behavior, Gillings School of Global Public Health, and Lineberger Comprehensive Cancer Center, University of North Carolina-Chapel Hill, Chapel Hill, North Carolina.

Vaccination is critical to controlling the COVID-19 pandemic, and health care providers play an important role in achieving high vaccination coverage (*1*). To examine the prevalence of report of a provider recommendation for COVID-19 vaccination and its association with COVID-19 vaccination coverage and attitudes, CDC analyzed data among adults aged ≥18 years from the National Immunization Survey-Adult COVID Module (NIS-ACM), a nationally representative cellular telephone survey. Prevalence of report of a provider recommendation for COVID-19 vaccination among adults increased from 34.6%, during April 22–May 29, to 40.5%, during August 29–September 25, 2021. Adults who reported a provider recommendation for COVID-19 vaccination were more likely to have received ≥1 dose of a COVID-19 vaccine (77.6%) than were those who did not receive a recommendation (61.9%) (adjusted prevalence ratio [aPR] = 1.12). Report of a provider recommendation was associated with concern about COVID-19 (aPR = 1.31), belief that COVID-19 vaccines are important to protect oneself (aPR = 1.15), belief that COVID-19 vaccination was very or completely safe (aPR = 1.17), and perception that many or all of their family and friends had received COVID-19 vaccination (aPR = 1.19). Empowering health care providers to recommend vaccination to their patients could help reinforce confidence in, and increase coverage with, COVID-19 vaccines, particularly among groups known to have lower COVID-19 vaccination coverage, including younger adults, racial/ethnic minorities, and rural residents.

NIS-ACM is a nationally representative household telephone survey of noninstitutionalized U.S. adults aged ≥18 years that uses a random-digit–dialed sample of cellular telephone numbers stratified by state and selected local jurisdictions (*2*). Data from five data collection periods were used for these analyses: April 22–May 29, May 30–June 26, June 27–July 31, August 1–August 28, and August 29–September 25, 2021. Response rates for these five periods ranged from 17.2% to 20.9%*; sample sizes ranged from 56,749 to 77,162, with an overall sample size of 340,543 participants. 

The survey assessed report of health care provider recommendation for COVID-19 vaccination,^†^ COVID-19 vaccination status,^§^ and attitudes toward vaccination. Attitudes toward vaccination were assessed by responses to four questions regarding 1) concern about COVID-19 infection (risk appraisal), 2) belief about the importance of COVID-19 vaccination (confidence), 3) belief about the safety of COVID-19 vaccination (confidence), and 4) belief about how many family and friends had received COVID-19 vaccination (social norms). These questions are based on the Behavioral and Social Drivers framework for increasing vaccine confidence^¶^ (*1*).

Prevalence of report of provider recommendation was assessed during April 22–September 25, 2021, and by period of data collection, sociodemographic characteristics,** U.S. Department of Health and Human Services (HHS) region,^††^ and jurisdiction.^§§^ Logistic regression was used to generate unadjusted and adjusted prevalence ratios (PRs and aPRs) of the association between the four attitudinal measures and both provider recommendation for COVID-19 vaccination and COVID-19 vaccination status. Adjusted analyses controlled for age group, sex, transgender identity, sexual orientation, race/ethnicity, education, income, insurance status, metropolitan statistical area (MSA),^¶¶^ U.S. Census region, comorbidity status,*** disability status,^†††^ essential worker status,^§§§^ and work or school COVID-19 vaccination requirement.^¶¶¶^ All variables assessed in this study were self-reported. The interaction between provider recommendation and each sociodemographic characteristic in predicting COVID-19 vaccination status was also assessed. The ecologic association between jurisdiction-level provider recommendation prevalence and jurisdiction-level vaccination coverage was also assessed using a Pearson correlation coefficient.

Data were analyzed using SAS (version 9.4; SAS Institute) and SUDAAN (version 11.0.3; RTI International). Results were weighted to represent the noninstitutionalized U.S. adult population aged ≥18 years and calibrated to COVID-19 vaccine administration data**** (*3*). For all analyses, statistical significance was defined as p<0.05. This activity was reviewed by the CDC and was conducted consistent with applicable federal law and CDC policy.^††††^

Prevalence of report of a provider recommendation for COVID-19 vaccines among adults increased from 34.6% during April 22–May 29 to 40.5% during August 29–September 25, 2021 (Table 1). From April 22–May 29 to August 29–September 25, report of provider recommendation ranged from 34.3% in HHS Region 10 to 42.7% in HHS Region 2 (Supplementary Table 1, https://stacks.cdc.gov/view/cdc/112307). Report of a provider recommendation was more common among adults aged ≥65 years (44.2%) than among those aged 18–29 years (28.3%); those with more than a college degree (45.6%) than among those with a high school education or less (33.5%); adults with annual household income of ≥$75,000 (39.8%) compared with those below the U.S. poverty threshold (36.9%); adults with health insurance (39.1%) compared with those without insurance (24.7%); adults who are essential health care workers (51.8%) compared with those in other essential work settings (32.1%–38.8%); and adults with comorbidities (50.4%) compared with those without (32.1%) (Table 1).

**TABLE 1:** Characteristics of adults who reported a health care provider recommendation for COVID-19 vaccination, by selected sociodemographic characteristics and associated factors — National Immunization Survey-Adult COVID Module, United States, April 22–September 25, 2021

Characteristic	Overall		Provider recommendation		
			Prevalence	Prevalence ratio	
	No.	% (95% CI)	% (95% CI)	Unadjusted (95% CI)	Adjusted* (95% CI)
**All adults**	**340,543**	**100.0**	**37.4 (37.1–37.7)**	—	—
**Period of data collection**					
Apr 22–May 29	77,162	20.0 (19.7–20.3)	34.6 (33.9–35.3)	Ref	Ref
May 30–Jun 26	56,749	20.0 (19.7–20.3)	35.8 (35.0–36.6)	1.03 (1.00–1.07)	1.03 (1.00–1.06)
Jun 27–Jul 31	73,512	20.0 (19.7–20.3)	37.6 (36.9–38.3)	1.09 (1.06–1.12)	1.08 (1.05–1.11)
Aug 1–Aug 28	63,193	20.0 (19.7–20.3)	38.6 (37.9–39.4)	1.12 (1.09–1.15)	1.10 (1.07–1.13)
Aug 29–Sep 25	73,426	20.0 (19.7–20.2)	40.5 (39.8–41.2)	1.17 (1.14–1.20)	1.14 (1.10–1.17)
**Age group, yrs**					
18–29	58,464	21.0 (20.7–21.3)	28.3 (27.6–29.0)	0.64 (0.62–0.66)	0.72 (0.69–0.74)
30–39	56,584	17.3 (17.1–17.6)	34.9 (34.2–35.7)	0.79 (0.77–0.81)	0.83 (0.80–0.85)
40–49	52,694	16.0 (15.7–16.2)	37.9 (37.1–38.7)	0.86 (0.83–0.88)	0.86 (0.83–0.89)
50–64	95,399	24.5 (24.2–24.8)	41.0 (40.4–41.7)	0.93 (0.91–0.95)	0.92 (0.89–0.94)
≥65	75,147	21.2 (20.9–21.5)	44.2 (43.5–45.0)	Ref	Ref
**Sex**					
Male	168,106	48.4 (48.1–48.8)	34.4 (34.0–34.9)	Ref	Ref
Female	173,190	51.6 (51.2–51.9)	40.3 (39.9–40.8)	1.17 (1.15–1.19)	1.07 (1.05–1.09)
**Transgender**					
Yes	13,287	4.5 (4.4–4.7)	36.5 (34.9–38.1)	0.98 (0.93–1.02)	1.01 (0.96–1.05)
No	309,379	95.5 (95.3–95.6)	37.4 (37.0–37.7)	Ref	Ref
**Sexual orientation**					
Heterosexual	298,486	92.6 (92.4–92.7)	37.5 (37.2–37.8)	Ref	Ref
Gay or lesbian	8,857	2.3 (2.2–2.4)	41.0 (39.0–43.1)	1.09 (1.04–1.15)	1.12 (1.06–1.17)
Bisexual	9,745	3.3 (3.1–3.4)	34.1 (32.3–35.9)	0.91 (0.86–0.96)	1.02 (0.96–1.07)
Other	5,654	1.9 (1.8–2.0)	36.3 (33.8–38.8)	0.97 (0.90–1.04)	1.06 (0.98–1.14)
**Race/Ethnicity**					
White, non-Hispanic	210,659	62.1 (61.8–62.4)	37.3 (36.9–37.7)	Ref	Ref
Black, non-Hispanic	40,610	12.0 (11.8–12.2)	38.4 (37.5–39.4)	1.03 (1.00–1.06)	1.02 (0.99–1.05)
Hispanic	43,420	17.2 (16.9–17.5)	37.2 (36.3–38.1)	1.00 (0.97–1.02)	1.09 (1.06–1.12)
Asian, non-Hispanic	17,859	4.2 (4.1–4.3)	40.0 (38.5–41.6)	1.07 (1.03–1.12)	1.11 (1.06–1.16)
American Indian or Alaska Native, non-Hispanic	8,319	1.3 (1.3–1.4)	38.8 (36.2–41.5)	1.04 (0.97–1.12)	1.13 (1.05–1.21)
Other or multiple races, non-Hispanic	12,865	3.2 (3.0–3.3)	36.2 (34.4–38.1)	0.97 (0.92–1.02)	1.03 (0.98–1.09)
**Educational level**					
High school or less	85,450	39.1 (38.7–39.4)	33.5 (33.0–34.1)	0.74 (0.72–0.75)	0.83 (0.80–0.85)
Some college	94,461	30.5 (30.2–30.9)	37.6 (37.0–38.2)	0.82 (0.80–0.84)	0.88 (0.86–0.90)
College graduate	85,631	19.2 (18.9–19.4)	40.7 (40.1–41.4)	0.89 (0.87–0.91)	0.96 (0.94–0.99)
Above college graduate	68,286	11.2 (11.1–11.4)	45.6 (44.8–46.4)	Ref	Ref
**Annual household income,^†^ USD**					
Below poverty	32,552	11.3 (11.1–11.5)	36.9 (35.9–37.9)	0.93 (0.90–0.95)	1.00 (0.96–1.03)
Above poverty and <$75,000	106,976	32.1 (31.8–32.5)	36.1 (35.5–36.7)	0.91 (0.89–0.93)	0.95 (0.93–0.97)
Above poverty and ≥$75,000	129,250	32.7 (32.4–33.0)	39.8 (39.3–40.3)	Ref	Ref
Unknown income	75,264	23.9 (23.6–24.2)	36.1 (35.5–36.8)	0.91 (0.89–0.93)	0.95 (0.92–0.97)
**Health insurance status**					
Insured	306,694	89.5 (89.3–89.7)	39.1 (38.7–39.4)	Ref	Ref
Not insured	27,335	10.5 (10.3–10.7)	24.7 (23.8–25.7)	0.63 (0.61–0.66)	0.75 (0.72–0.78)
**Essential worker status** ^§^					
Essential health care	36,028	9.1 (8.9–9.3)	51.8 (50.8–52.9)	1.40 (1.37–1.43)	1.38 (1.35–1.42)
School and child care	12,789	2.9 (2.8–3.0)	38.8 (37.1–40.5)	1.05 (1.00–1.09)	1.02 (0.97–1.07)
Other frontline	24,835	8.4 (8.2–8.6)	32.3 (31.2–33.4)	0.87 (0.84–0.90)	1.02 (0.98–1.06)
Other essential	39,597	12.5 (12.2–12.7)	32.1 (31.2–33.0)	0.87 (0.84–0.89)	1.00 (0.97–1.04)
Not an essential worker	228,472	67.2 (66.9–67.5)	37.1 (36.7–37.5)	Ref	Ref
**MSA** ^¶^					
MSA, principal city	106,173	29.1 (28.8–29.4)	38.6 (38.0–39.2)	Ref	Ref
MSA, nonprincipal city	172,259	57.2 (56.9–57.5)	37.4 (37.0–37.8)	0.97 (0.95–0.99)	0.96 (0.94–0.98)
Non-MSA	65,610	13.7 (13.5–13.9)	34.9 (34.1–35.7)	0.90 (0.88–0.93)	0.92 (0.89–0.95)
**U.S. Census region**					
Northeast	70,694	17.4 (17.3–17.6)	42.1 (41.4–42.7)	Ref	Ref
Midwest	54,434	20.8 (20.5–21.0)	36.7 (35.9–37.4)	0.87 (0.85–0.89)	0.93 (0.90–0.95)
South	70,212	23.8 (23.5–24.0)	36.3 (35.6–37.1)	0.86 (0.84–0.89)	0.88 (0.86–0.91)
West	126,934	38.0 (37.8–38.3)	36.0 (35.5–36.5)	0.86 (0.84–0.87)	0.92 (0.90–0.94)
Comorbidities**					
Yes	102,135	29.2 (28.9–29.5)	50.4 (49.8–51.1)	1.57 (1.54–1.60)	1.47 (1.44–1.50)
No	237,651	70.8 (70.5–71.1)	32.1 (31.7–32.5)	Ref	Ref
**Disability status** ^††^					
Yes	30,864	9.7 (9.5–9.9)	44.9 (43.8–46.0)	1.23 (1.20–1.26)	1.11 (1.07–1.14)
No	312,280	90.3 (90.1–90.5)	36.6 (36.3–36.9)	Ref	Ref
**Work or school requirement** ^§§^					
Yes	43,949	10.8 (10.6–11.0)	49.8 (48.8–50.7)	1.39 (1.36–1.42)	1.32 (1.29–1.36)
No/Other	297,453	89.2 (89.0–89.4)	35.9 (35.5–36.2)	Ref	Ref

**Abbreviations:** MSA = metropolitan statistical area; Ref = referent group; USD = U.S. dollars.

*Adjusted for age group, sex, transgender identity, sexual orientation, race/ethnicity, education, income, insurance status, MSA, U.S. Census region, comorbidity status, disability status, and essential worker status.

^†^ Household income is derived from the number of persons reported in the household, the reported household income, and the 2020 U.S. Census poverty thresholds.

^§^ Essential worker status was defined based on the following questions: “Are you a frontline or essential worker according to your state or region?” and “In what location or setting do you currently work?” Essential worker groups were categorized as “essential healthcare,” “school and childcare,” “other frontline,” “other essential,” and “nonessential.” Nonessential could include both employed and unemployed individuals.

^¶^ MSA status was determined based on household reported city and county of residence and was grouped into three categories: MSA principal city (urban), MSA nonprincipal city (suburban), and non-MSA (rural). MSAs and principal cities were as defined by the U.S. Census Bureau (https://www.census.gov/programs-surveys/metro-micro.html). Non-MSA areas include urban populations not located within an MSA as well as completely rural areas.

** Comorbidity status was ascertained by the following question: “Do you have a health condition that may put you at higher risk for COVID-19?”

^††^ Disability status was ascertained by the following question: “Do you have serious difficulty seeing, hearing, walking, remembering, making decisions, or communicating?”

^§§^ Work or school requirement was assessed by the following question: “Does your work or school require you to get a COVID-19 vaccine?” Response options were yes, no, or unemployed/not applicable. Responses for “no” and “not applicable” were combined into one category.

Adults who had received a provider recommendation were more likely to have received ≥1 dose of COVID-19 vaccine (77.6%) than were those who did not receive a recommendation (61.9%) (aPR = 1.12) (Table 2). Analyses of the interaction between provider recommendation and sociodemographic characteristics on vaccine receipt found that provider recommendation was associated with higher likelihood of receipt of ≥1 COVID-19 vaccine dose among most subgroups, with highest aPR for younger adults (aged 18–29 and 30–39 years; aPR = 1.22), non-Hispanic American Indian or Alaska Native adults (aPR = 1.19), adults living in rural areas (aPR = 1.18), adults living in the West (aPR = 1.17) or Midwest (aPR = 1.15), and adults who did not have a school or work COVID-19 vaccination requirement (aPR = 1.15).

**TABLE 2:** Association of report of a health care provider recommendation for COVID-19 vaccination and receipt of ≥1 COVID-19 vaccine dose, overall and by selected sociodemographic characteristics — National Immunization Survey-Adult COVID Module, United States, April 22–September 25, 2021

Characteristic	Receipt of ≥1 COVID-19 vaccine dose, % (95% CI)			
	Provider recommendation		Prevalence ratio	
	Yes	No	Unadjusted	Adjusted*
**Overall**	**77.6 (77.1–78.1)**	**61.9 (61.5–62.3)**	**1.25 (1.24–1.27)**	**1.12 (1.11–1.14)**
**Age group, yrs**				
18–29	63.3 (61.9–64.7)	45.4 (44.5–46.3)	1.39 (1.35–1.44)	1.22 (1.18–1.26)
30–39	68.7 (67.4–70.1)	51.1 (50.1–52.2)	1.34 (1.31–1.38)	1.22 (1.18–1.25)
40–49	74.5 (73.2–75.8)	57.5 (56.4–58.6)	1.30 (1.26–1.33)	1.19 (1.16–1.22)
50–64	80.9 (80.0–81.8)	70.0 (69.1–70.8)	1.16 (1.14–1.18)	1.08 (1.06–1.10)
≥65	91.0 (90.3–91.7)	87.4 (86.7–88.1)	1.04 (1.03–1.05)	1.03 (1.01–1.04)
**Sex**				
Male	78.9 (78.2–79.5)	64.2 (63.6–64.9)	1.23 (1.21–1.24)	1.12 (1.11–1.14)
Female	78.9 (78.2–79.5)	64.2 (63.6–64.9)	1.23 (1.21–1.24)	1.15 (1.13–1.16)
**Race/Ethnicity**				
White, non-Hispanic	80.5 (79.9–81.1)	63.1 (62.6–63.7)	1.27 (1.26–1.29)	1.17 (1.15–1.19)
Black, non-Hispanic	69.0 (67.5–70.5)	56.4 (55.1–57.7)	1.22 (1.19–1.26)	1.07 (1.03–1.10)
Hispanic	74.0 (72.6–75.4)	60.2 (58.9–61.4)	1.23 (1.20–1.26)	1.09 (1.06–1.12)
Asian, non-Hispanic	88.3 (86.2–90.5)	87.1 (85.5–88.7)	1.01 (0.98–1.05)	1.01 (0.96–1.05)
American Indian or Alaska Native, non-Hispanic	69.1 (64.7–73.5)	46.6 (43.1–50.1)	1.48 (1.34–1.64)	1.19 (1.09–1.30)
Other or multiple races, non-Hispanic	67.8 (64.6–71.1)	48.7 (46.3–51.1)	1.39 (1.30–1.49)	1.15 (1.07–1.24)
**Essential worker^†^**				
Essential health care	81.8 (80.6–83.1)	68.3 (66.7–69.8)	1.20 (1.17–1.23)	1.12 (1.08–1.15)
School and child care	84.9 (82.4–87.4)	78.5 (76.4–80.5)	1.08 (1.04–1.13)	1.04 (0.99–1.10)
Other frontline	67.0 (64.8–69.1)	52.2 (50.7–53.7)	1.28 (1.23–1.34)	1.10 (1.07–1.14)
Other essential	68.2 (66.5–69.9)	50.2 (49.0–51.4)	1.36 (1.31–1.41)	1.15 (1.11–1.18)
Not an essential worker	79.3 (78.7–79.9)	64.5 (63.9–65.0)	1.23 (1.22–1.24)	1.15 (1.13–1.16)
**MSA** ^§^				
MSA, principal city	78.1 (77.2–79.0)	65.4 (64.6–66.2)	1.19 (1.17–1.21)	1.10 (1.08–1.12)
MSA, nonprincipal city	78.6 (78.0–79.3)	62.6 (62.0–63.2)	1.26 (1.24–1.27)	1.14 (1.12–1.16)
Non-MSA	71.7 (70.3–73.0)	52.2 (51.1–53.3)	1.37 (1.33–1.41)	1.18 (1.14–1.21)
**U.S. Census region**				
Northeast	81.8 (80.9–82.8)	71.9 (71.0–72.8)	1.14 (1.12–1.16)	1.08 (1.05–1.10)
Midwest	75.4 (74.2–76.6)	57.9 (56.9–59.0)	1.30 (1.27–1.33)	1.15 (1.12–1.18)
South	81.3 (80.2–82.4)	67.8 (66.8–68.8)	1.20 (1.18–1.22)	1.11 (1.09–1.14)
West	74.1 (73.3–74.9)	55.9 (55.3–56.6)	1.32 (1.30–1.35)	1.17 (1.15–1.19)
**Comorbidities** ^¶^				
Yes	83.5 (82.8–84.2)	71.2 (70.3–72.1)	1.17 (1.15–1.19)	1.15 (1.13–1.17)
No	74.1 (73.4–74.7)	59.3 (58.8–59.8)	1.25 (1.23–1.27)	1.13 (1.11–1.14)
Work or school requirement**				
Yes	88.2 (87.1–89.2)	85.7 (84.6–86.8)	1.03 (1.01–1.05)	1.03 (1.01–1.05)
No/Other	75.8 (75.2–76.3)	59.6 (59.2–60.1)	1.27 (1.26–1.28)	1.15 (1.14–1.17)

**Abbreviation:** MSA = metropolitan statistical area.

*Adjusted for age group, sex, transgender identity, sexual orientation, race/ethnicity, education, income, insurance status, MSA, U.S. Census region, comorbidity status, disability status, and essential worker status.

^†^ Essential worker status was defined based on the following questions: “Are you a frontline or essential worker according to your state or region?” and “In what location or setting do you currently work?” Essential worker groups were categorized as “essential healthcare,” “school and childcare,” “other frontline,” “other essential,” and “nonessential.” Nonessential may include both employed and unemployed individuals.

^§^ MSA status was determined based on household reported city and county of residence and was grouped into three categories: MSA principal city (urban), MSA nonprincipal city (suburban), and non-MSA (rural). MSAs and principal cities were as defined by the U.S. Census Bureau (https://www.census.gov/programs-surveys/metro-micro.html). Non-MSA areas include urban populations not located within an MSA as well as completely rural areas.

^¶^ Comorbidity status was ascertained by the following question: “Do you have a health condition that may put you at higher risk for COVID-19?”

** Work or school requirement was assessed by the following question: “Does your work or school require you to get a COVID-19 vaccine?” Response options were yes, no, or unemployed/not applicable. Responses for “no” and “not applicable” were combined into one category.

Report of a provider recommendation was associated with concern about COVID-19 (aPR = 1.31), confidence that COVID-19 vaccines are important to protect oneself (aPR = 1.15), confidence that COVID-19 vaccination was very or completely safe (aPR = 1.17), and perception that many or all of their family and friends had received COVID-19 vaccination (aPR = 1.19) (Supplementary Table 2, https://stacks.cdc.gov/view/cdc/112308).

In the jurisdiction-level correlation analysis, COVID-19 vaccination coverage was higher among persons living in jurisdictions with higher prevalence of provider recommendation (correlation coefficient = 0.66) (Figure) (Supplementary Table 1, https://stacks.cdc.gov/view/cdc/112307). For example, in Wyoming, prevalence of report of a provider recommendation was 30.1%, and COVID-19 vaccination coverage was 51.2%, whereas in Puerto Rico, prevalence of provider recommendation was 50.5%, and COVID-19 vaccination coverage was 77.5%.

**FIGURE:**
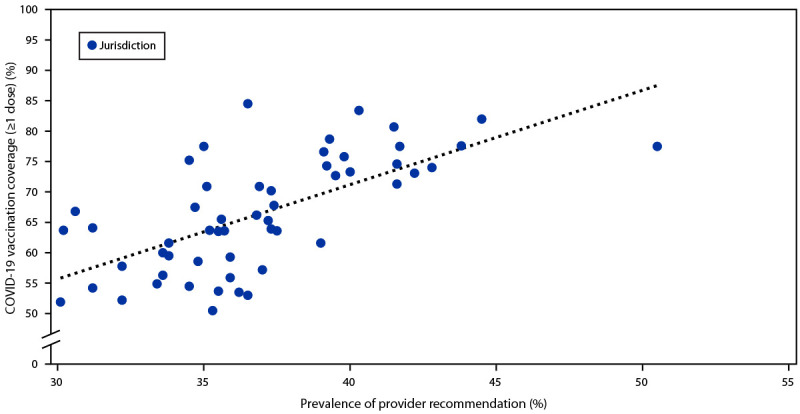
Correlation of prevalence of report of health care provider recommendation and COVID-19 vaccination coverage (≥1 dose) among 53 jurisdictions,* by jurisdiction — National Immunization Survey Adult-COVID Module, United States, April 22–September 25, 2021 * Sample correlation coefficient = 0.66.

## Discussion

Health care providers are among the most trusted sources of information on safety and effectiveness of vaccines, and their recommendations are strongly associated with vaccination acceptance (*4*,*5*). This study found that provider recommendation was associated with higher likelihood of getting vaccinated, as well as higher likelihood of having concerns about COVID-19, confidence that vaccines are important to protect oneself from COVID-19, confidence that COVID-19 vaccines are very or completely safe, and perception that many or all of one’s family and friends had received COVID-19 vaccine. The findings from an ecologic analysis also suggest that jurisdictions’ prevalence of provider recommendations was positively associated with jurisdiction-level COVID-19 vaccination coverage.

Similar to report of a provider recommendation for influenza vaccine, which was 33.0% in 2016 (*6*), report of a provider recommendation for vaccination against COVID-19 remains low. Approximately less than one half of participants nationwide reported receiving a provider recommendation, with <40% of persons in rural areas and in some jurisdictions reporting a provider recommendation. These patterns mirror known patterns in disparities in health insurance coverage, financial barriers to care, and the use of wellness visits and checkups; as a result, lower access to health care might reduce the opportunity for interactions with trusted providers (*7*).

As COVID-19 vaccine availability in primary care settings increases and patients become eligible for additional or booster doses, provider recommendation will continue to serve an important role in motivating individual patient vaccination acceptance and completion (*8*). Health care systems and medical practices can benefit from procedures that build patient and provider confidence in COVID-19 vaccination and strengthen the capacity of health care providers to have conversations about vaccines, address misinformation, and provide tailored information to patients. As trusted sources of medical information, providers have the opportunity to clearly recommend COVID-19 vaccines as a main strategy for preventing serious health outcomes from COVID-19 (*9*).

The findings in this study are subject at least six limitations. First, response rates were low (approximately 20%), but consistent with other NIS surveys (*2*). Bias in estimates might remain after weighting for household nonresponse and incomplete sample frame (households with only landline or no telephone service were excluded). Second, vaccination receipt, provider recommendation, and other characteristics (e.g., essential worker status or medical conditions) were self-reported and subject to recall and misclassification bias. For example, the question on medical conditions could have been interpreted by some survey respondents as medical conditions that place them at higher risk for exposure to COVID-19; however, a secondary analysis of a follow-up question on condition type found that approximately 75% indicated a medical condition associated with higher risk for severe COVID-19. Moreover, survey weights were calibrated to COVID-19 vaccine administration data (*3*) to mitigate possible bias from incomplete sample frame, nonresponse, and misclassification of vaccination status. Third, the survey did not measure health care provider visits, so a low number of reports of provider recommendation could be due to limited access to health care providers. Fourth, attitudes might have changed over time with changes in the Advisory Committee on Immunization Practices vaccination recommendations or the emergence of the highly transmissible SARS-CoV-2 B.1.617.2 (Delta) variant (*10*). Fifth, the categorization of attitudinal measures was conservative (e.g., classifying someone who reported “somewhat safe” as not believing COVID-19 vaccination is safe), which might have underestimated observed associations. Finally, the survey is cross-sectional; thus, causal relationships cannot be inferred, including the association between beliefs about COVID-19 vaccination and report of a provider recommendation. For example, providers might be more likely to recommend vaccines to persons who express more concerns or who seem more receptive to vaccination; alternatively, these persons might be more likely to remember and report receiving a provider recommendation. In addition, causality between the ecological association of provider recommendation at the jurisdiction level and vaccination coverage cannot be inferred.

Health care providers are uniquely positioned to provide COVID-19 vaccination recommendations, and it is important that they continue to promote COVID-19 vaccination to eligible persons. This is particularly important among groups with lower COVID-19 vaccination coverage, including younger adults, racial/ethnic minorities, persons with lower education and income, and rural residents. Empowering health care providers to recommend COVID-19 vaccines at every visit and reducing barriers to health care access could increase confidence in vaccines and COVID-19 vaccination coverage.

**Summary**

**What is already known about this topic?**

COVID-19 vaccination is critical to controlling the COVID-19 pandemic; health care providers play an important role in achieving high vaccination coverage.

**What is added by this report?**

Adults who reported a provider COVID-19 vaccination recommendation were more likely to have been vaccinated, to be concerned about COVID-19, to have confidence that COVID-19 vaccines are important and safe, and to perceive that family and friends had been vaccinated.

**What are the implications for public health practice?**

A health care provider recommendation for COVID-19 vaccines at every visit could increase coverage and confidence in vaccines, particularly among groups with lower COVID-19 vaccination coverage, including younger adults, racial/ethnic minorities, and rural residents.

## Footnotes

* Calculated according to the American Association for Public Opinion Research type 3 response rate. https://www.aapor.org/AAPOR_Main/media/publications/Standard-Definitions20169theditionfinal.pdf

† Report of a provider recommendation was assessed with the following question: “Has a doctor, nurse, or other health professional ever recommended that you get a COVID-19 vaccine?”

§ COVID-19 vaccination status was assessed by response to the following question: “Have you received at least one dose of a COVID-19 vaccine?”

¶ Concern about COVID-19 infection was assessed by the following questions: “How concerned are you about getting COVID-19? Would you say you are: not at all concerned; a little concerned; moderately concerned; or very concerned?” Adults who responded with moderately or very concerned were categorized as being concerned about COVID-19 infection. Beliefs about the importance of vaccination was assessed by the following question: “How important do you think getting a COVID-19 vaccine is to protect yourself against COVID-19? Would you say it is not at all important, a little important, somewhat important, or very important?” Adults who responded with somewhat or very important were categorized as believing that vaccination is important for protection against COVID-19. Beliefs about the safety of COVID-19 vaccines was assessed by the following questions: “How safe do you think a COVID-19 vaccine is for you? Would you say not at all safe; somewhat safe; very safe; or completely safe?” Adults who responded with very or completely safe were categorized as having beliefs that vaccine is safe. Finally, social norms were assessed by the following questions: “If you had to guess, about how many of your family and friends have received a COVID-19 vaccine? Would you say none; some; many; or almost all?” Adults who responded with many or almost all were categorized as having family and friends who were all or mostly vaccinated.

** Sociodemographic demographic characteristics were age group, sex, transgender identity, sexual orientation, race/ethnicity, education, income, insurance status, MSA, U.S. Census region, comorbidity status, disability status, essential worker status, and work or school requirement.

†† *Region 1:* Connecticut, Maine, Massachusetts, New Hampshire, Rhode Island, and Vermont; *Region 2:* New Jersey, New York, Puerto Rico, and the U.S. Virgin Islands; *Region 3:* Delaware, District of Columbia, Maryland, Pennsylvania, Virginia, and West Virginia; *Region 4*: Alabama, Florida, Georgia, Kentucky, Mississippi, North Carolina, South Carolina, and Tennessee); *Region 5*: Illinois, Indiana, Michigan, Minnesota, Ohio, and Wisconsin; *Region 6*: Arkansas, Louisiana, New Mexico, Oklahoma, and Texas; *Region 7:* Iowa, Kansas, Missouri, and Nebraska; *Region 8:* Colorado, Montana, North Dakota, South Dakota, Utah, and Wyoming; *Region 9:* Arizona, California, Hawaii, Nevada, American Samoa, Northern Mariana Islands, Federated States of Micronesia, Guam, Marshall Islands, and Palau; *Region 10:* Alaska, Idaho, Oregon, and Washington.

§§ Fifty-three jurisdictions were defined as the 50 states; Washington, DC; and two U.S. territories (Puerto Rico and U.S. Virgin Islands). The U.S. territory of Guam was excluded because of a limited data collection period.

¶¶ MSA status was determined based on household reported city and county of residence and was grouped into three categories: MSA principal city (urban), MSA nonprincipal city (suburban), and non-MSA (rural). MSAs and principal cities were as defined by the U.S. Census Bureau. https://www.census.gov/programs-surveys/metro-micro.html

*** Comorbidity status was ascertained by the following question: “Do you have a health condition that may put you at higher risk for COVID-19?” This was followed by the question, “Can you tell me what that is?” Responses to this second question indicate ≥75% of respondents interpreted the question as medical conditions that have been associated with higher risk of severe COVID-19.

††† Disability status was ascertained by the following question: “Do you have serious difficulty seeing, hearing, walking, remembering, making decisions, or communicating?”

§§§ Essential worker status was defined based on the following questions: “Are you a frontline or essential worker according to your state or region?” and “In what location or setting do you currently work?” Response options were 1) health care (e.g., hospital, doctor, dentist or mental health specialist office, outpatient facility, long-term care, home health care, pharmacy, or medical laboratory); 2) social service (e.g., child, youth, family, elderly, or disability services); 3) preschool or daycare; 4) K–12 school; 5) other schools and instructional settings (e.g., college, university, professional, business, technical or trade school, driving school, test preparation, or tutoring); 6) first response (e.g., police or fire protection, or emergency relief services); 7) death care (e.g., funeral home, crematory, or cemetery); 8) correctional facility (e.g., jail, prison, detention center, or reformatory); 9) food and beverage store (e.g., grocery store, warehouse club, supercenters, convenience store, specialty food store, or bakery); 10) agriculture, forestry, fishing, or hunting; 11) food manufacturing facility (e.g., meat processing, produce packing, or food or beverage manufacturing); 12) nonfood manufacturing facility (e.g., metals, equipment and machinery, or electronics); 13) public transit (e.g., bus, commuter rail, subway, or school bus); 14) United States Postal Service; and 15) other. Essential worker groups who responded with 1, 2, and 7 were categorized as “essential health care,” 3–5 were categorized as “school and childcare,” 6 and 8–14 were categorized as “other frontline,” and 15 were categorized as “other essential,” and those who answered “no” to the first question were categorized as “not an essential worker.” Nonessential could include both employed and unemployed persons.

¶¶¶ Work or school requirement was assessed by the following question: “Does your work or school require you to get a COVID-19 vaccine?” Response options were “yes,” “no,” or “unemployed/not applicable.” Responses for “no” and “not applicable” were combined into one category.

**** Survey weights were calibrated to the COVID-19 vaccine administration data by jurisdiction, age group, and sex.

†††† 45 C.F.R. part 46.102(l)(2), 21 C.F.R. part 56; 42 U.S.C. Sect. 241(d); 5 U.S.C. Sect. 552a; 44 U.S.C. Sect. 3501 et seq.
